# Spontaneous Intracranial Hypotension Presenting as a "Pseudo-Chiari 1

**DOI:** 10.7759/cureus.1034

**Published:** 2017-02-16

**Authors:** Ali S Haider, Suraj Sulhan, Ian T Watson, Dean Leonard, Eliel N Arrey, Umair Khan, Phu Nguyen, Kennith F Layton

**Affiliations:** 1 Texas A&M College of Medicine; 2 UT Houston Medical School, Memorial Hermann; 3 School of Medicine, St. Georges University; 4 Department of Radiology, Baylor University Medical Center

**Keywords:** arnold – chiari type 1 malformation, epidural blood patch, posterior fossa decompression, spontaneous intracranial hypotension

## Abstract

Spontaneous intracranial hypotension (SIH) is classified as a decrease in cerebrospinal fluid (CSF) pressure secondary to a CSF leakage and consequent descent of the brain into the foramen magnum. Diagnosing SIH can be difficult due to its overlapping findings with Arnold-Chiari type 1 Malformation (CM1) where the cerebellar tonsils herniate into the foramen magnum. The similarity of both conditions calls for a more reliable imaging technique to localize the CSF leak which could narrow the differential diagnosis and aid in choosing the correct treatment. Here, we present a case of a 28-year-old female, ten weeks post-partum with symptoms similar to SIH. MRI of the brain was remarkable for tonsillar herniation below the foramen magnum. Literature was reviewed for additional neuroradiology techniques that would aid in narrowing our differential diagnosis. Interestingly, computed tomography-, digital subtraction-, and magnetic resonance myelography with intrathecal gadolinium are the preferred techniques for diagnosis of high flow and low flow CSF leaks, respectively. These modalities further aid in choosing the correct treatment while avoiding complications. Literature suggests that treatment for CM1 involves posterior fossa decompression, whereas the mainstay of treatment for SIH involves an epidural blood patch (EBP). Thus, our patient was treated with an EBP and recovered without complication.

## Introduction

Spontaneous intracranial hypotension (SIH) is a neurological condition characterized by cerebral spinal fluid (CSF) pressure below 60 mmH2O with descent of the brain into the foramen magnum [1]. Recent neuroimaging studies have shown CSF leakage to be the probable etiology [2]. Diagnosis of SIH is supported by magnetic resonance imaging (MRI), indicating the displacement of the brainstem and causing occipital headache and meningism. These symptoms and findings also overlap with Arnold-Chiari type 1 malformation (CM1), another condition characterized by displacement of the cerebellar tonsils through the foramen magnum. We present a rare and interesting case of occipital headaches with cerebellar displacement into the foramen magnum and discuss the importance of additional imaging to narrow the differential diagnosis. Informed consent was obtained from the patient for this study.

## Case presentation

**Figure 1:**
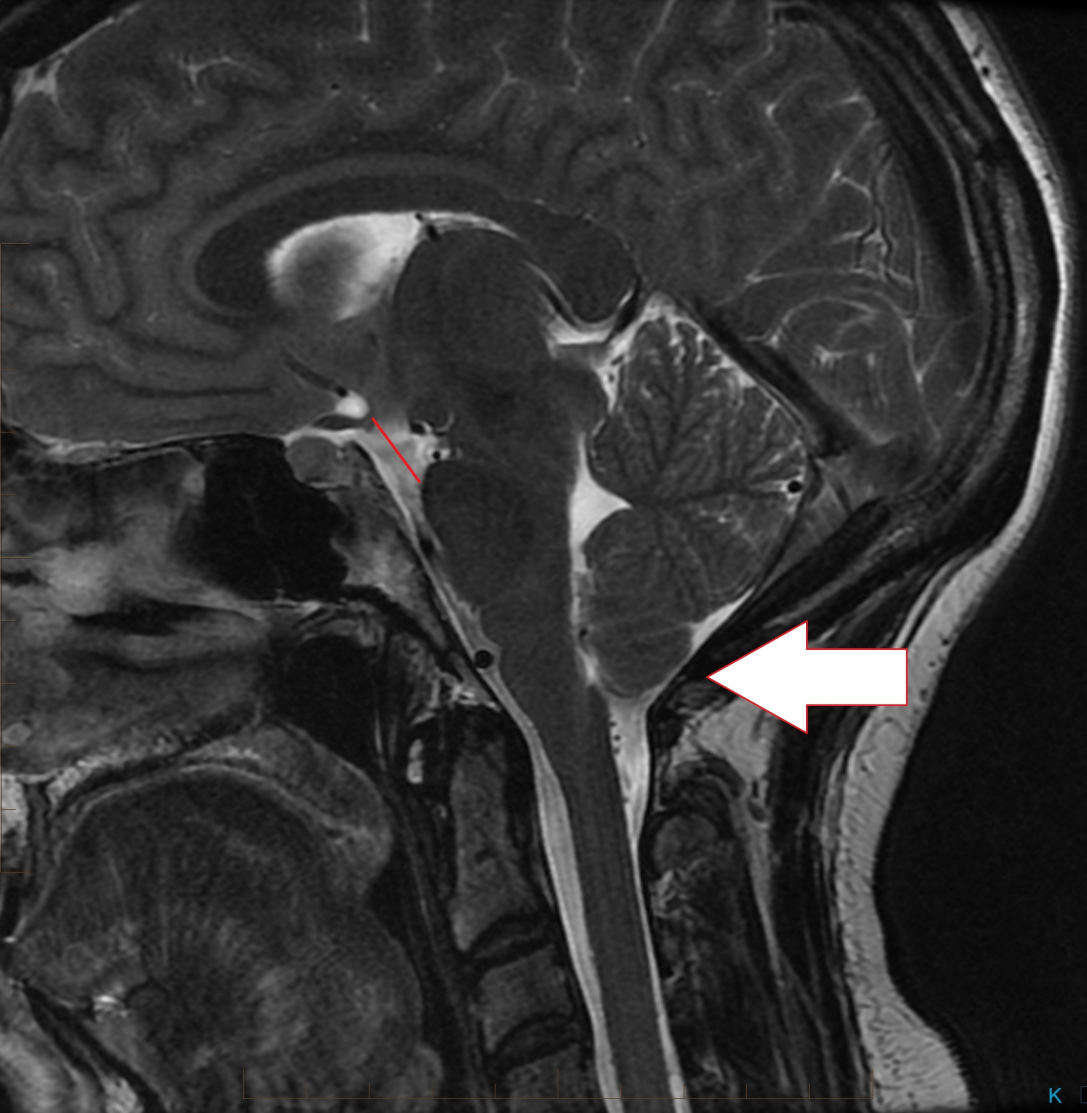
Sagittal T2 brain MRI demonstrating low-lying cerebellar tonsils with a decrease in the mamillo-pontine distance and draping of the optic chiasm over the dorsum sellae

**Figure 2:**
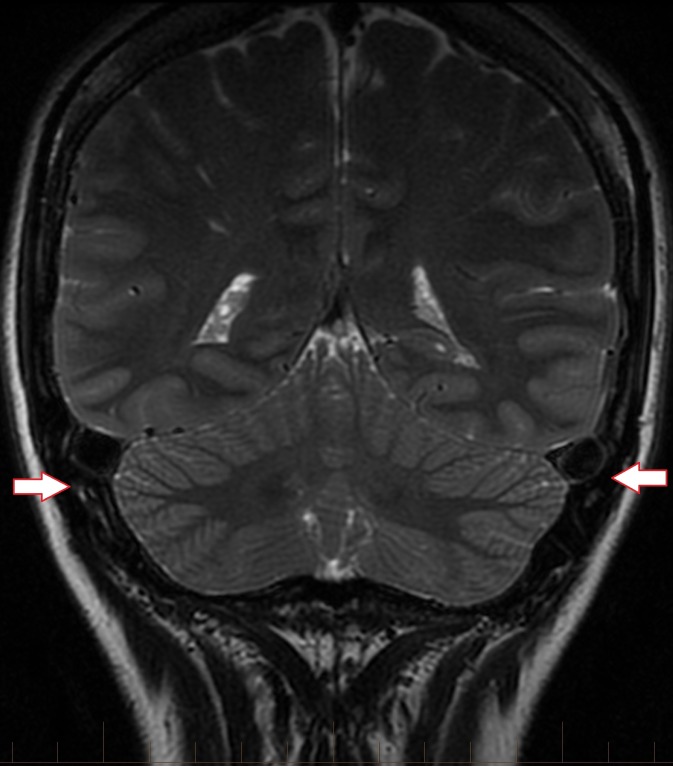
Coronal T2 brain MRI revealing an engorged appearance of the bilateral transverse dural venous sinuses

A 28-year-old female, ten weeks post-partum with a two-year history of headaches at the posterior skull base presented after the headaches increased in severity over the last seven weeks. The headaches were noticeably worse when crouching or lifting. The patient had received epidural anesthesia during childbirth ten weeks ago. Brain magnetic resonance imaging (MRI) without contrast showed extension of the cerebellar tonsils 8 mm below the foramen magnum with crowding of the brainstem at the level of the foramen. In addition, there was a 4 mm decrease in the mamillo-pontine distance, and sagging of the posterior fossa and diencephalon with draping of the optic chiasm over the dorsum sellae and posterior clinoids. The dural venous sinuses were enlarged (Fig. 1, 2). CSF flow study suggested that there was perturbation of flow dynamics at the skull base.

**Figure 3:**
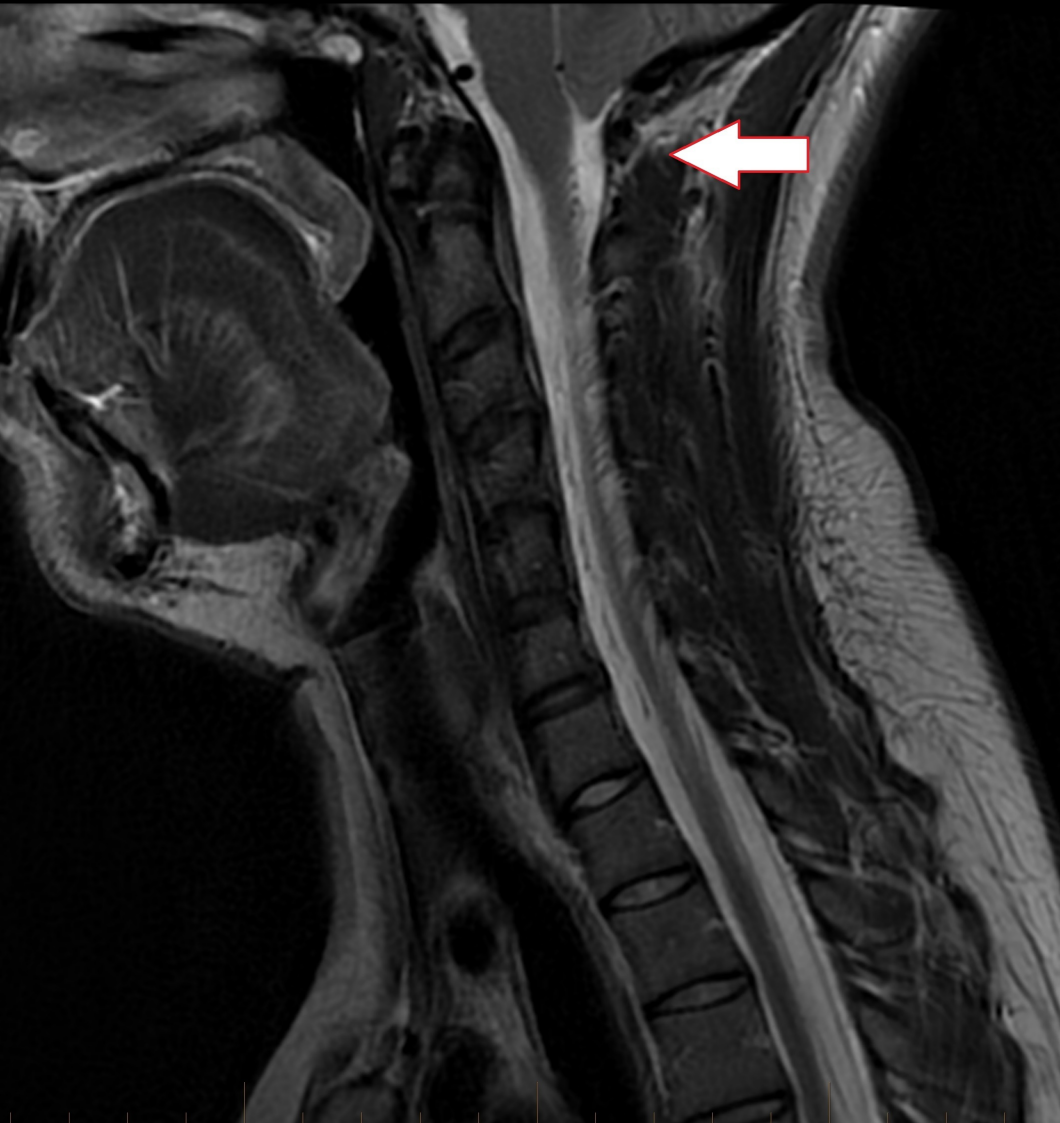
Sagittal T2 cervical spine MRI of mid-line revealing inferior descent of the cerebellar tonsils below the level of the foramen magnum down to the level of the C1 posterior arch

**Figure 4:**
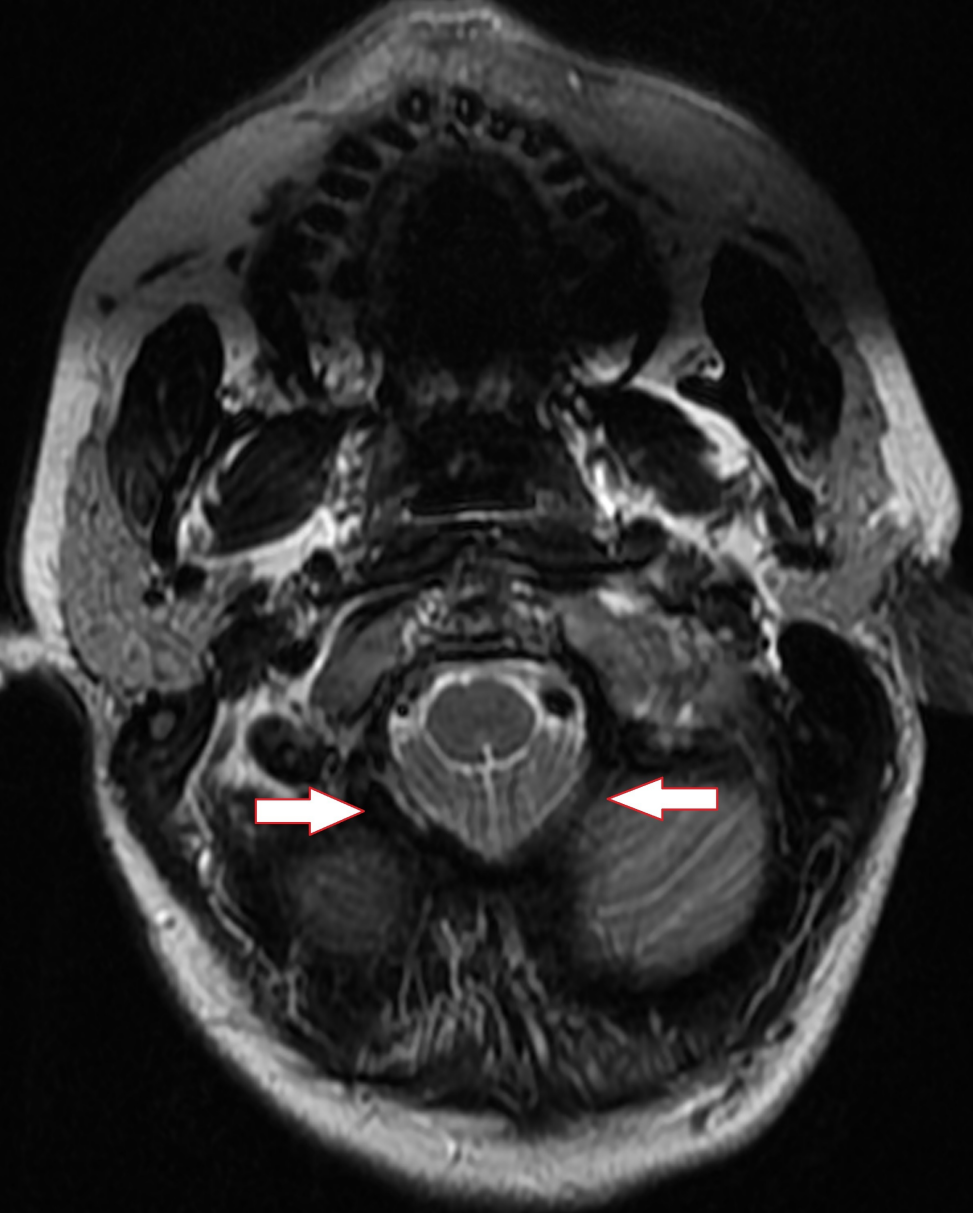
Axial T2 cervical spine MRI at the skull base demonstrating crowding of the foramen magnum and inferior descent of the cerebellar tonsils

MRI of the cervical spine without contrast confirmed the inferior positioning of the cerebellar tonsils by 7-8 mm (Fig. 3, 4). In the absence of additional imaging a pseudo-Chiari secondary to intracranial hypotension was highly suspected without eliminating the possibility of a true CM1. A brain MRI with contrast and an MRI of the thoracic and lumbar spine were recommended to look for the typical smooth dural thickening and possible leakage of spinal CSF but the patient was lost to follow-up.

## Discussion

SIH is described as CSF pressure less than 60 mmH2O and descent of the brain into the foramen magnum. The fall in CSF pressure is secondary to a CSF leak, likely due to a recent lumbar puncture or epidural anesthesia [3-4]. CSF leakage can result in venous engorgement resulting in pachymeningeal enhancement on MRI. Since CSF provides protective buoyancy to intracranial structures, a leak can cause the brain to descend and increased traction of pain-sensitive structures. Patients may present with postural occipital headache, nausea, vomiting, radiculopathy, tinnitus, vertigo, nuchal rigidity, photophobia and cranial nerve palsies [2,4-6]. Recognition of a prominent inferior intercavernous sinus may assist in diagnosing SIH, which presents as a rounded structure at the floor of the sellae seen in 50% of patients with SIH [6]. Difficulty in diagnosing SIH is due to overlapping findings with CM1, a congenital syndrome characterized by herniation of the cerebellar tonsils into the foramen magnum without brainstem involvement. The pathogenesis remains unclear, but herniation of the cerebellar tonsils can lead to pain, weakness, dysphagia and sensory disturbances. An occipital headache is one of the most common presenting symptoms occurring in 15%-98% of patients, commonly accentuated by postural change or exertion [3,7]. Puget, et al., however, described a pseudo-Chiari tonsillar herniation in SIH due to a CSF leak with no association to syringomyelia, but rather, pachymeningeal enhancement [4]. This highlights a key difference in the two syndromes. The overlap of symptomology between SIH and CM1 calls for the reliance on additional imaging to localize a CSF leak as seen in SIH. Computed tomography (CT) myelography is the preferred diagnostic modality to detect initial CSF leaks followed by dynamic CT myelography to differentiate high-flow from low-flow leaks [8]. CT myelography or digital subtraction myelography are specific for high-flow leaks, whereas magnetic resonance myelography with intrathecal gadolinium is preferred for low-flow leaks [8]. Treatment for SIH involves rest, caffeine, fluid supplementation, or an epidural blood patch (EBP). EBP is the current mainstay of treatment and can be targeted to the specific site of a CSF leak on imaging or delivered blindly into the lumbar region [6]. One retrospective, non-randomized series showed that 87% of patients who received a single targeted EBP experienced a benefit and 100% after receiving two EBP procedures [4,6-9].

## Conclusions

Though patients with SIH present with findings similar to those seen in a CM1, advances in neuroradiology and myelographic techniques can narrow the differential diagnosis and ultimately support the correct treatment choice for the patient.
